# Single-Gene Overexpression of Pigeon Pea (*Cajanus cajan*) *CcSBPase* or *CcSHMT1* and Multigene Overexpression of *CcSBPase* with *CcSTN7* Do Not Impact Physiological Responses to Water Deficit Stress in Tobacco (*Nicotiana tabacum*)

**DOI:** 10.3390/plants15192963

**Published:** 2026-09-29

**Authors:** Malizukiswe Vincent Vacu, Bianke Loedolff, Shaun Peters

**Affiliations:** 1 Institute for Plant Breeding and Biotechnology, Faculty of AgriSciences, Stellenbosch University, Stellenbosch 7602, South Africa; bianke@sun.ac.za (B.L.); swpeters@sun.ac.za (S.P.); 2 Department of Plant Production, Soil Science, and Agricultural Engineering, School of Agricultural and Environmental Sciences, University of Limpopo, Polokwane 0727, South Africa

**Keywords:** water deficit stress, abiotic stress, photosynthesis, *SBPase*, *SHMT1*, *STN7*

## Abstract

Drought stress is one of the environmental stress factors exacerbated by climate change. It causes water deficit stress, leading to economic yield losses. Its impact is largely felt in semi-arid and arid regions, often occupied by resource-limited farmers. This study aimed to determine whether transgenic tobacco plants improved for photosynthesis performance to enhance plant yield perform better than wild-type (WT) untransformed plants under induced water deficit stress conditions. In doing so, transgenic tobacco (*Nicotiana tabacum*) plants with genes that encode sedoheptulose-1,7-bisphosphatase (*SBPase*), serine/threonine protein kinase-7 (*STN7*), and serine hydroxymethyltransferase-1 (*SHMT1*) enzymes from pigeon pea (*Cajanus cajan*) were exposed to water deficit stress by withholding water for 15 days. The transgenic lines used were composed of single-gene T3 transgenic lines and F2 generation multigene overexpressing lines. To determine the response to water deficit stress, specific physiological parameters were recorded on days 0, 8, and 15 of withholding water supply. These parameters included leaf relative water content (RWC), chlorophyll fluorescence (Fv/Fm), and stomatal conductance, accompanied by daily growth substrate moisture measurements (soil moisture). The results show a decline in photosynthesis in WT and all transgenic lines, suggesting that there is no improvement in tolerance to water deficit stress. This is observed through the decline in stomatal conductance and Fv/Fm from day 8 to 15 after withholding the water supply. Based on these observations, we conclude that the overexpression of pigeon pea *SBPase* and *SHMT1*, as well as the simultaneous overexpression of *SBPase* with *STN7*, do not improve physiological responses to induced water deficit stress in the tobacco model plant.

## 1. Introduction

As the human population increases and climate variability intensifies, more emphasis must be placed on improving food production. At the heart of this is the need to adopt sustainable practices that reduce the environmental burden associated with high plant water demand and the overreliance on high fertiliser and agrochemical inputs. One of the proposed and largely explored sustainable approaches for plant improvement has been the engineering of photosynthesis. The resulting photosynthetically improved plants have been reported to boost yields by more efficiently using natural resources such as light and carbon dioxide (CO_2_) [1,2,3,4,5].

Although success in yield improvement has been reported using photosynthesis manipulation in both model and crop plants [3,4,6,7,8,9,10], investigation of the response to drought-induced water deficit stress among improved genotypes is necessary. During drought stress, leaf water status decreases, leading to reduced electron transport and ultimately lower generation of ATP and NADPH for the Calvin–Benson (CB) cycle [11]. This further affects the rate of CO_2_ fixation in the CB cycle. Functional analysis of cotton (*Gossypium hirsutum* L.) *SBPase* revealed an upregulated gene expression in response to induced drought stress [12], further suggesting that the function of SBPase is closely linked to tolerance to abiotic stress as previously reported in rice (*Oryza sativa*) under salt stress [13] and in tomato (*Solanum lycopersicum*) under chilling stress experiments [6,14,15]. SBPase alleviates the bottleneck in the CB cycle during abiotic stress by accelerating the regeneration of ribulose-1,5-bisphosphate (RuBP), thereby maintaining higher CO_2_ assimilation. This ensures that the photosynthesis capacity of the plant remains optimal, with the biosynthesis of soluble sugars, which are important for plant abiotic stress tolerance, serving as osmoprotectants, antioxidants, and signalling molecules [16]. During water deficit stress, stomatal openings close due to abscisic acid (ABA) upregulation, limiting CO_2_ assimilation and leading to Rubisco-catalysed oxygenation of RuBP. When this happens, it leads to elevated photorespiration and overreduction of electron transport, which favours reactive oxygen species (ROS) hyperaccumulation [17]. Photorespiration converts 2-phosphoglycolate (2PG) to 3-phosphoglycerate (3PGA), which is used as a carbon source for growth and development; as such, photorespiration has been viewed as a key pathway in facilitating drought stress tolerance [18]. An important step during the 2PG-3PGA conversion is a glycine–serine reversible conversion facilitated by serine hydroxymethyltransferase-1 (SHMT1) enzyme in most higher plants.

SHMT1 is well documented for its role in facilitating the reversible conversion of glycine to serine, an important step in the recovery of CO_2_ during photorespiration [19,20,21,22]. In alfalfa (*Medicago sativa*), the functional analysis of SHMT family genes revealed that among other members, *SHMT1* expression increases in response to salt and drought stress [20]. The absence of *SHMT1* activity in *Arabidopsis thaliana shmt1* is associated with sensitivity to salinity and drought stress, underscoring the importance of *SHMT1* in plant abiotic stress tolerance [23].

The electron transport flow is negatively affected by drought stress due to hyperaccumulation of ROS, which disrupts the photosystem reaction centres. The result is inefficient electron transport, which affects the overall growth and development of plants. The serine/threonine protein kinase-7 (*STN7*) facilitates the phosphorylation of light-harvesting complex 2 (LHC11) to maintain efficient electron transport, which is important for optimal photosynthesis [24,25]. Investigation of the response of rapeseed (*Brassica napus*) seedlings to drought stress identified *STN7* as a drought-specific protein, as it was responsive to drought but not to salinity stress [26]. That study showed the potential of STN7 as a phosphorylating protein in mediating drought stress. There is no literature available on the analysis of *STN7* overexpressing lines under water deficit stress to determine whether this candidate gene induces water deficit stress tolerance.

A multigene transgenic approach has been reported to improve plant performance more than single-gene-expressing lines in rice [27], tobacco [8], and Arabidopsis [28]. The approach offers a combinatory benefit of candidate genes. In this study, we report on the performance of the *CcSBPase*–*CcSTN7* transgene combination under water deficit stress.

To date, there has been no functional characterisation of *CcSBPase*, *CcSHMT1*, and *CcSTN7* in response to water deficit stress. Thus, to understand the role of these genes, current research was conducted using tobacco model plants with heterologous expression of the *Cajanus cajun* mitochondrial *SHMT1*, chloroplast *SBPase*, and the combination of *SBPase* with the electron transport *STN7*. Observing the relative water content (RWC), stomatal conductance, and chlorophyll fluorescence (Fv/Fm) in the respective transgenic lines revealed no improvement in drought induced water deficit stress tolerance compared with WT.

## 2. Materials and Methods

### 2.1. Plant Materials and Growth Conditions

The plant materials used were wild type (WT) *Nicotiana tabacum* cv Samsun and transgenic lines generated with the overexpression of *Cajanus cajan SBPase* (*CcSBPase*, herein referred to as SB L2 and L3), and *SHMT1* (*CcSHMT1*, herein referred to as SH L1 and L2) driven by a dual *35S* promoter. The seeds of WT and T3 transgenic lines were used in this study. Another set of seeds was the F2 generation (herein referred to as SBST) of multigene transgenic lines resulting from lines derived from a cross between T2 transgenic lines that overexpress *CcSBPase* and *CcSTN7*, respectively (Figure S1, Supplementary Materials). The lines of the cross between *CcSBPase* and *CcSHMT1* were not used in this experiment due to significant fold change expression inconsistencies observed from F1 to F2 generation (Figures S2 and S3, Supplementary Materials). The materials were generated from a separate study conducted within the Institute for Plant Breeding and Biotechnology, Stellenbosch University.

Seeds from all plant lines were germinated in vitro using Petri dishes. After twenty days of in vitro growth in tissue culture, seedlings at growth stage 1002 [29] were transferred to 15 cm pots with 2:1:1 peat: sand: vermiculite. The plants were grown under 16 h of light at 25 ± 1 °C, 8 h of dark, 18 ± 1 °C, and 60% relative humidity. Light was supplied by OrbitX Neptune LED 150 W IP65 lights (OrbitX, Paarl, South Africa), providing varying average light throughout the day [120 µmol m^−2^s^−1^ at the beginning of the day (3 h); 230–333 µmol m^−2^s^−1^ (2 h); 440–450 µmol m^−2^s^−1^ at the middle of the day (for 6 h); 230–333 µmol m^−2^s^−1^ (2 h); and 120 µmol m^−2^s^−1^ at the end of the day (3 h)].

Plant nutrition was supplied in liquid form using 1.5 g/L of hydroponic fertiliser (Hygrotech©, Stellenbosch, South Africa) with 1.5 g/L of calcium nitrate after seven days of seedling transfer to the growth mixture. The pots were irrigated regularly until growth stage 1105 to 1107, marked by 5 to 7 unfolded leaves, where water deficit stress was imposed by withholding water for 15 days. The pots were randomly rotated every second day.

### 2.2. Stomatal Conductance and Chlorophyll Fluorescence

At the growth stage 1105 to 1107 on day zero of water deficit stress, the source leaf (leaf order number 3 counting from the bottom) was dark-adapted for 25 min. Chlorophyll fluorescence (Fv/Fm) was measured using an OS-30p + fluorometer (OPTI-SCIENCES, Hudson, MH, USA) in WT and transgenic lines. Measurements were taken on three biological replicates per line each day of data collection (0, 8, 15 days). Stomatal conductance (mmol.m^−2^m^−1^) was measured on the same leaf three times per replicate using the Decan Leaf Porometer SC-1 (Decagon Devices, Pullman, WA, USA). A similar procedure was performed on days eight and fifteen.

### 2.3. Soil Moisture and Relative Water Content

From day 0, soil moisture content (m^3^/m^3^) was measured every day (3 readings per pot) until day 15 using HydroSense II (Campbell Scientific, Logan, UT, USA). The sampling of the relative water content of the leaf was taken from the source leaf used for Fv/Fm and stomatal conductance. The relative water content of the leaves was determined as previously described [30]. The percentage relative water content (RWC) was calculated using the formula: RWC = (FW − DW)/(TW − DW) × 100.

### 2.4. Statistical Analysis

The study was carried out in a completely randomised design, with three biological replicates for each harvest interval. Data were analysed using GraphPad Prism version 5, with Student’s paired *t*-test used to compare the means of WT with the respective transgenic lines. Data are presented as means ± standard error of means (SEM) with statistically significant differences denoted by *p* ≤ 0.05 (*), ≤0.01 (**), ≤0.001 (***).

## 3. Results

### 3.1. Soil Moisture Content and RWC as a Measure of Plant Water Status

To assess the response to water deficit stress, we have exposed WT and transgenic tobacco lines to days 0, 8, and 15 without watering. Unstressed plants (day 0) did not show a significant difference in RWC (Figure 1). This trend continued until day 15, except for the multigene transgenic line SBST L5, which showed significantly reduced RWC compared to WT on day 8 (Figure 1C). The steady decrease in RWC corresponded to a decrease in soil moisture content. Soil moisture content was measured daily, and no significant differences were observed between WT and any of the transgenic lines under study (Figure 1D).

The decrease in soil moisture content and RWC affected the phenotype of WT and transgenic lines. The lack of water in the soil resulted in visible wilting of all plants, without a visible difference between WT and transgenic lines (Figure 2 and Figure 3). Multigene overexpression of *CcSBPase* and *CcSTN7* also did not show any signs of better performance (Figure 3).

### 3.2. The Stomatal Conductance and Chlorophyll Fluorescence as Indicators of Photosynthesis Performance

To gain insight into changes in photosynthesis, we have measured the stomatal conductance and chlorophyll fluorescence of photosystem II (PSII) (Fv/Fm) (Figure 4). Compared to WT, under unstressed conditions (day 0), the *35S::CcSHMT1* lines exhibited significantly higher stomatal conductivity and Fv/Fm (Figure 4A,D). This indicated a significantly improved photosynthesis performance in both *35S::CcSHMT1* transgenic lines. However, on day 8 of water deficit, both stomatal conductance and Fv/Fm decreased significantly in these lines, indicating sensitivity to water deficit stress. Although no significant differences were observed on day zero, on day eight, there was a significant decrease in stomatal conductance in *35S::CcSBPase* (SBL2) and in both multigene transgenic lines compared to WT. The decrease was observed through day fifteen for both the stomatal conductance and Fv/Fm. The Fv/Fm declined from an average of 0.80 (on days 0 and 8) to 0.70 (on day 15) on WT, SBST, and *CcSBPase* lines. A varied response was observed in the *CcSHMT1* lines, with L1 averaging 0.69 and L2 0.65 on day 15, while day 8 averaged 0.77 (Figure 4D–F).

## 4. Discussion

Water deficit stress caused by drought stress is one of the detrimental effects of climate variability. It affects mainly rainfed agriculture. Water deficit stress results in a decrease in economic crop yields [11], which puts pressure on food security. This requires urgent intervention to develop drought-tolerant high-yielding varieties. The engineering of the CB cycle *SBPase* has been shown to confer tolerance to abiotic stress factors such as chilling and high-temperature stress in cultivated crops [6,14,31]. Similarly, previous studies have identified photorespiratory *SHMT1* as an important factor in the induction of tolerance to abiotic stress (drought, high light, and salt stress) and tolerance to biotic stress (pathogen attack) in plants [23,32]. These previous reports support the hypothesis that *SBPase* and *SHMT1* are key targets for combating abiotic and biotic stressors.

However, in contrast to previous studies, in the current study, using tobacco transgenic lines that overexpress *CcSBPase* and *CcSHMT1*, we report a decrease in leaf water status as measured by RWC. This is accompanied by a decrease in the parameters related to photosynthesis. Under drought stress, RWC decreases, leading to cell dehydration and, consequently, reducing the water potential in the apoplast [11]. The resulting low RWC under drought stress is directly associated with low crop yields [33]. These effects are linked to physiological disruptions caused by water deficit stress, which plants experience when exposed to drought stress. The decrease in RWC observed in transgenic lines and WT indicates that *CcSBPase* and *CcSHMT1* have no potential to minimise yield losses due to water deficit stress. The decrease in RWC occurs at the same time as a decrease in key components of efficient photosynthesis, as measured by the decrease in the stomatal conductance and chlorophyll fluorescence of PSII (Fv/Fm). The decrease in stomatal conductance is an indication of stomatal closure to reduce water loss, which, in turn, reduces the photosynthesis rate by limiting CO_2_ uptake [34]. Inhibition of photosynthesis under abiotic stress conditions is commonly assessed by measuring Fv/Fm [29,35,36], in which a reduction in Fv/Fm is associated with impaired PSII function [17], highlighting sensitivity to stress. The Fv/Fm provides a measure of leaf health, which is compromised under water deficit stress when ROS accumulate in cytoplasmic organelles, resulting in reduced photosynthesis activity. The observed minimum Fv/Fm values of 0.65–0.69 in *CcSHMT1* lines on day 15 indicate that PSII function was still active [37] but decreased faster than WT, which recorded an average of 0.70, suggesting that the reaction centre is sensitive to drought stress over time.

There is no available literature linking *STN7* overexpression to abiotic and biotic stress responses. The upregulation of native STN7 protein in rapeseed suggested a possible role for this protein in drought stress tolerance, even though the plants suffered from exposure to drought stress [26]; hence, the need to understand its role when overexpressed. The *STN7* kinase enzyme is well established for the phosphorylation of LHCII to improve adaptation to changing light conditions [25,38,39]. The phosphorylation of LHCII balances the energy excitation between PSII and photosystem I (PSI), ensuring photosynthesis efficiency. If left unchecked, the excess energy leads to overreduction of electron transport, favouring ROS accumulation. Therefore, the phosphorylation of LCHII is important for maintaining energy balance and preventing excess energy, which can negatively affect electron transport [40]. In our preliminary study, the *35S::CcSTN7* transgenic tobacco lines showed high sensitivity to terminal water deficit conditions within a few days. Similarly, in a multigene overexpression approach with *CcSBPase*, we observe no improvement in tolerance to water deficit stress. Based on analysis of some of the key physiological parameters measured in this study, our findings agree with recent findings that the function of *STN7* kinase is limited to phosphorylation of LHCII, with no direct beneficial effect on mitigating water deficit stress beyond WT performance [39]. It is worth noting that the study focused only on the seedling stage and on controlled environmental conditions. Additional studies in different model plants and cultivated crops at various growth stages could be conducted to further analyse the effects of *CcSBPase*, *CcSHMT1*, and *CcSTN7* on water deficit stress induced by drought or soil salinity. The inclusion of metabolite markers would further enhance understanding of the responses of *CcSBPase*, *CcSHMT1*, and *CcSTN7* transgenic lines to water deficit stress. The effects of *CcSBPase*, *CcSHMT1*, and *CcSTN7* on water deficit stress tolerance could be further examined under field conditions to assess the performance of the respective transgenic lines relative to WT.

## Figures and Tables

**Figure 1:**
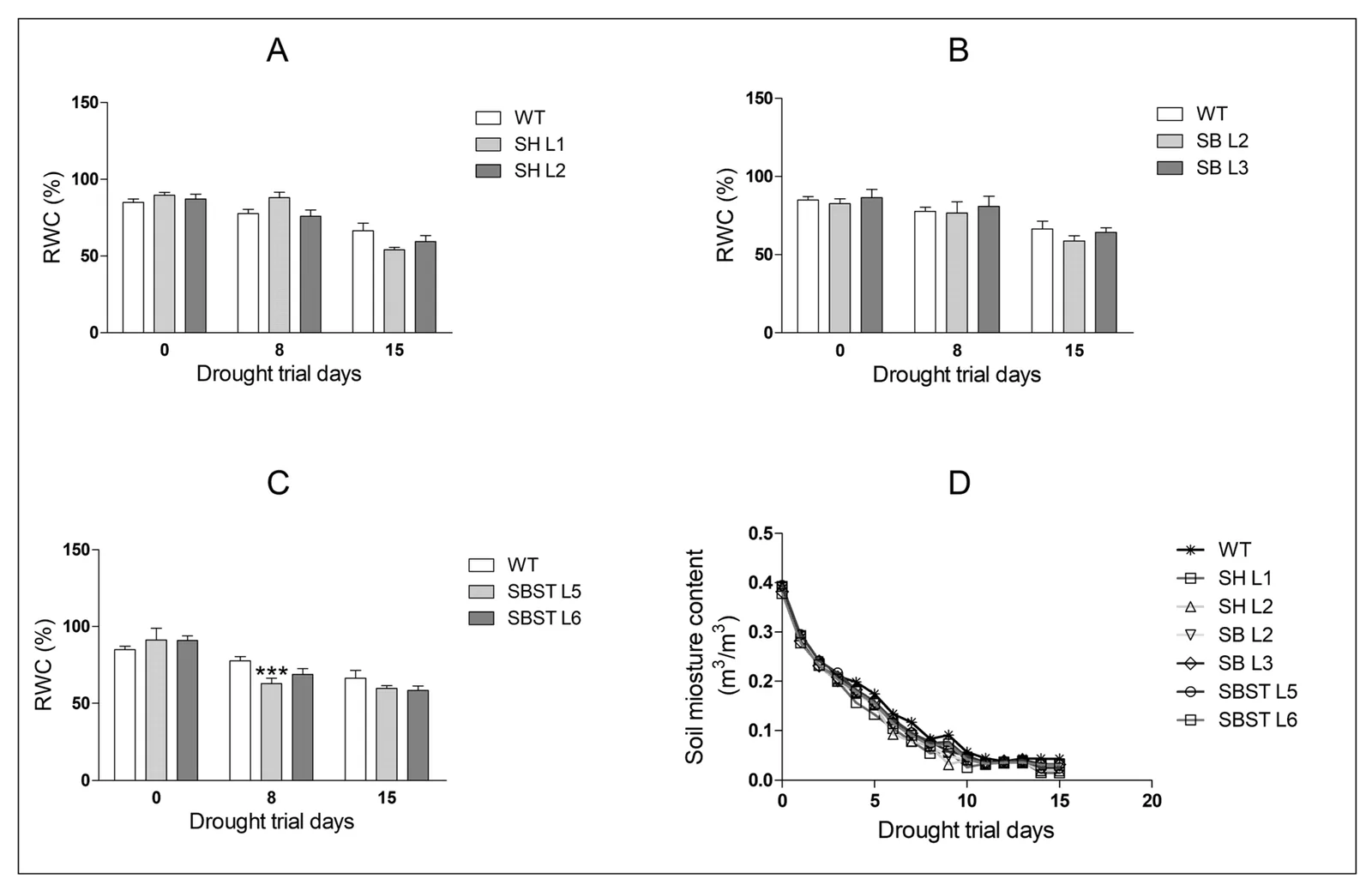
The leaf relative water content (RWC) in WT and transgenic lines (**A**–**C**) and soil moisture content (**D**) taken during the 15-day water deficit trial. WT stands for wild type; SH L1 and SH L2 represent *35S::CcSHMT1* lines; SB L2 and SB L3 represent *35S::CcSBPase* lines; SBST L5 and SBST L6 represent lines of the cross *35S::CcSBPase* X *35S::CcSTN7*. Values represent means ± SEM (*n* = 6). Data are presented as means ± standard error of means (SEM) with statistically significant differences denoted by *p* ≤ 0.001 (***).

**Figure 2:**
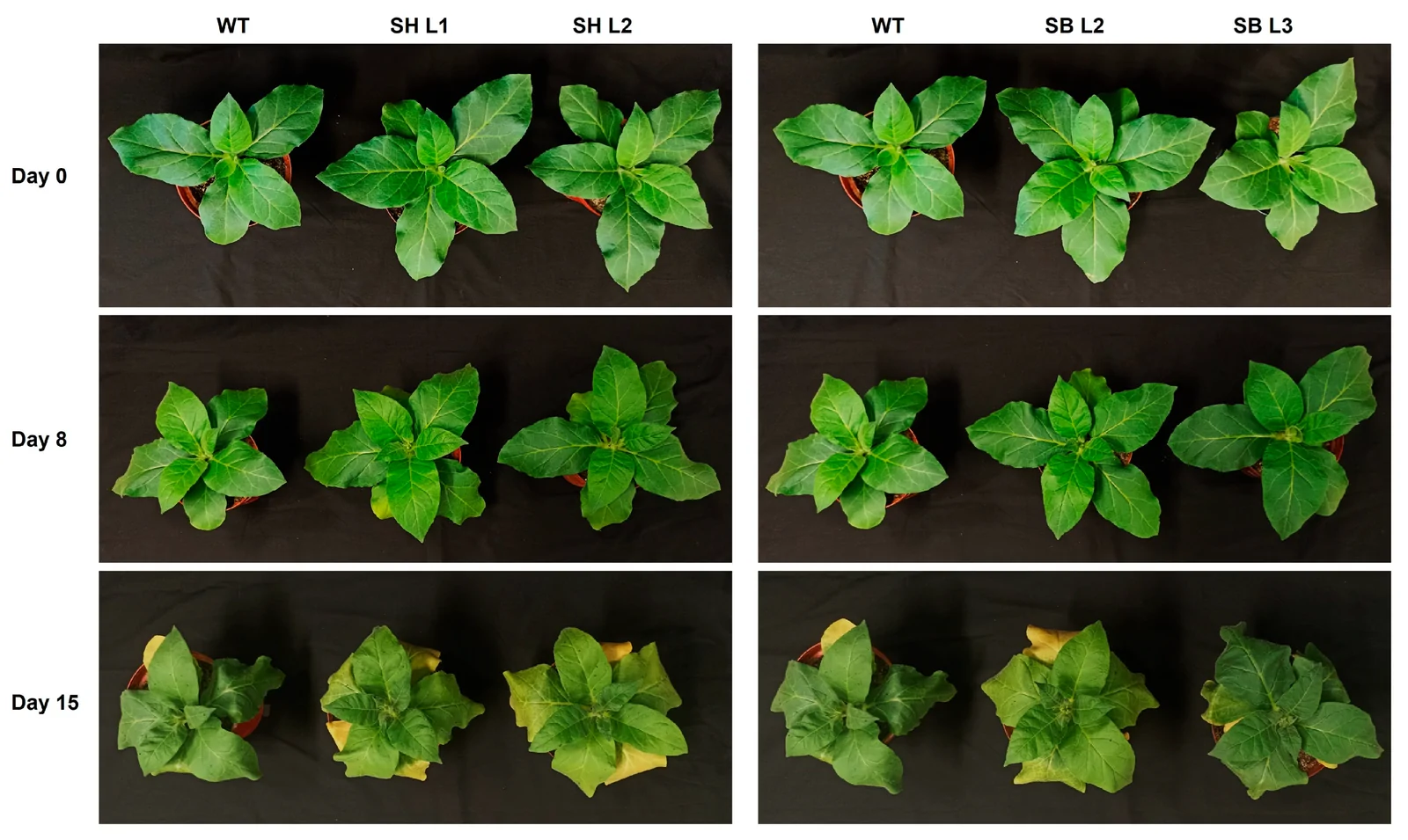
The phenotype of wild type (WT) and single-gene overexpressing lines (SH L1 and SH L2; SB L2 and SB L3) under study. Images were taken on days 0, 8, and 15 of exposure to the water deficit trial.

**Figure 3:**
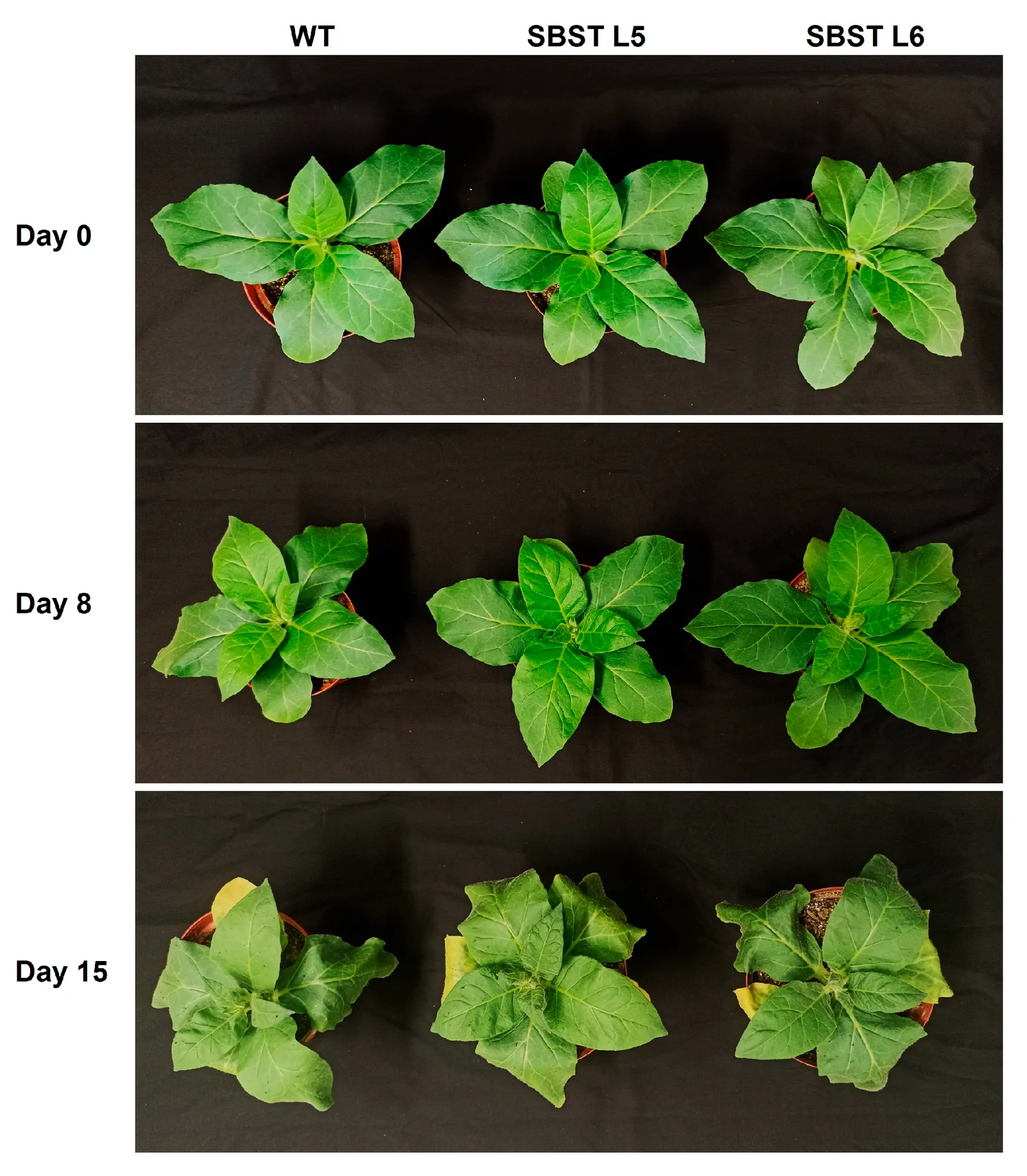
The phenotype of wild type and multigene transgenic lines (SBST L5 and L6). The images were taken on days 0, 8, and 15 of exposure to the water deficit trial.

**Figure 4:**
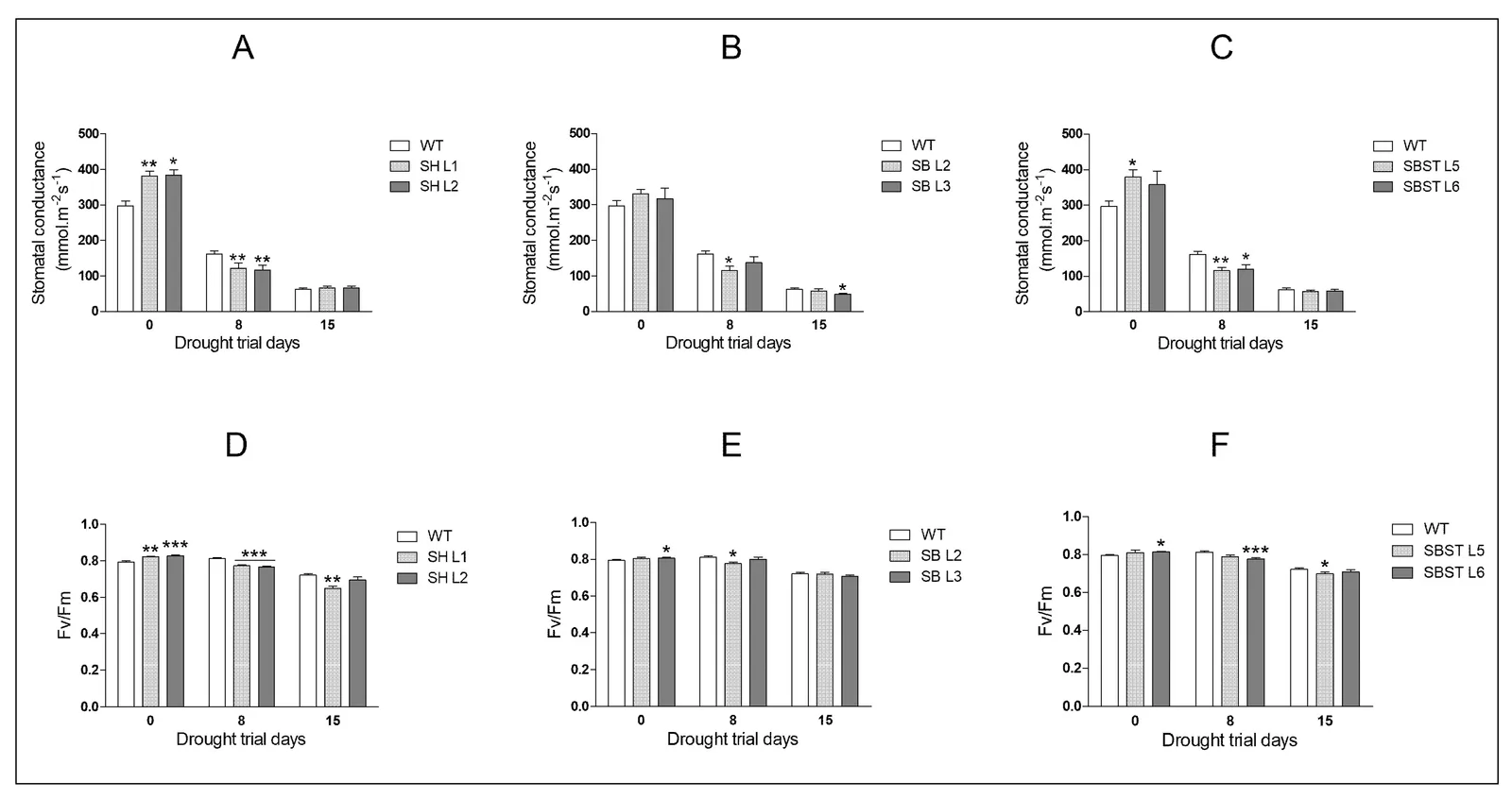
The stomatal conductance (**A**–**C**) and chlorophyll fluorescence (Fv/Fm) (**D**–**F**) of the *35S::CcSHMT1* lines (SH L1 and L2), the 35*S::CcSBPase* lines (SB L2 and L3), and the multigene transgenic lines (SBST L5 and L6), each line compared to wild type (WT). Measurements were taken on days 0, 8, and 15 during the 15-day water deficit trial. Data are presented as means ± standard error of means (SEM) with statistically significant differences denoted by *p* ≤ 0.05 (*), ≤0.01 (**), ≤0.001 (***).

## Data Availability

The original contributions presented in this study are included in the article/Supplementary Materials. Further inquiries can be directed to the corresponding author.

## Supplementary Materials

The following supporting information can be downloaded at: https://www.mdpi.com/article/10.3390/plants15192963/s1, Figure S1. The RT-qPCR results of T2 *N.tabacum* transgenic lines. (A) The fold change data normalized against *HIST2H3A*, and (B) normalized against *HSC70-1*. Note: WT-Wild Type; N.D.—Not detected; Lines with asterisks (*) indicate a significant difference at *p* < 0.05. A paired student t-test was used to compare the means of each transgenic line to WT. Figure S2. A RT-qPCR representation of F2 generation. (i) The fold change data normalized against the *HIST2H3A* reference gene, and (ii) normalised against *HSC70-1* for each crossing event. Note: WT-Wild Type; N.D.- Not detected; Lines with asterisks (*) indicate a significant difference at *p* < 0.05. A paired student t-test was used to compare the means of each transgenic line to WT. Figure S3. A RT-qPCR representation of the *N.tabacum* F1 generation resulting from a cross between (i) *35S*::*CcSHMT1/N.tabacum* L4(♀) X T2 *35S*::*CcSTN7/N. tabacum* L3(♂); (ii) *35S*::*CcSHMT1/N.tabacum* L2(♀) X L6 of F1 CcSBPase X CcSTN7 (♂); and (iii) *35S*::*CcSBPase/N.tabacum* Line 2(♀) X *35S:*: *CcSTN7/N.tabacum* Line 3(♂). The data is log2 fold change expression normalized against three reference genes. Note: WT-Wild Type; N.D.—Not detected; Lines with asterisks (*) indicate a significant difference at *p* < 0.05. A paired student t-test was used to compare the means of each transgenic line to WT.
