# Supplementary material for: Single-Gene Overexpression of Pigeon Pea (Cajanus cajan) CcSBPase or CcSHMT1 and Multigene Overexpression of CcSBPase with CcSTN7 Do Not Impact Physiological Responses to Water Deficit Stress in Tobacco (Nicotiana tabacum)
Source: Plants (Basel). 2026 Sep 29;15(19):2963. doi: 10.3390/plants15192963 (PMC13666972; doi:10.3390/plants15192963)
Supplement: Supplementary file 1 [file plants-15-02963-s001.zip › plants-4394143-supplementary.pdf]

Supplementary materials

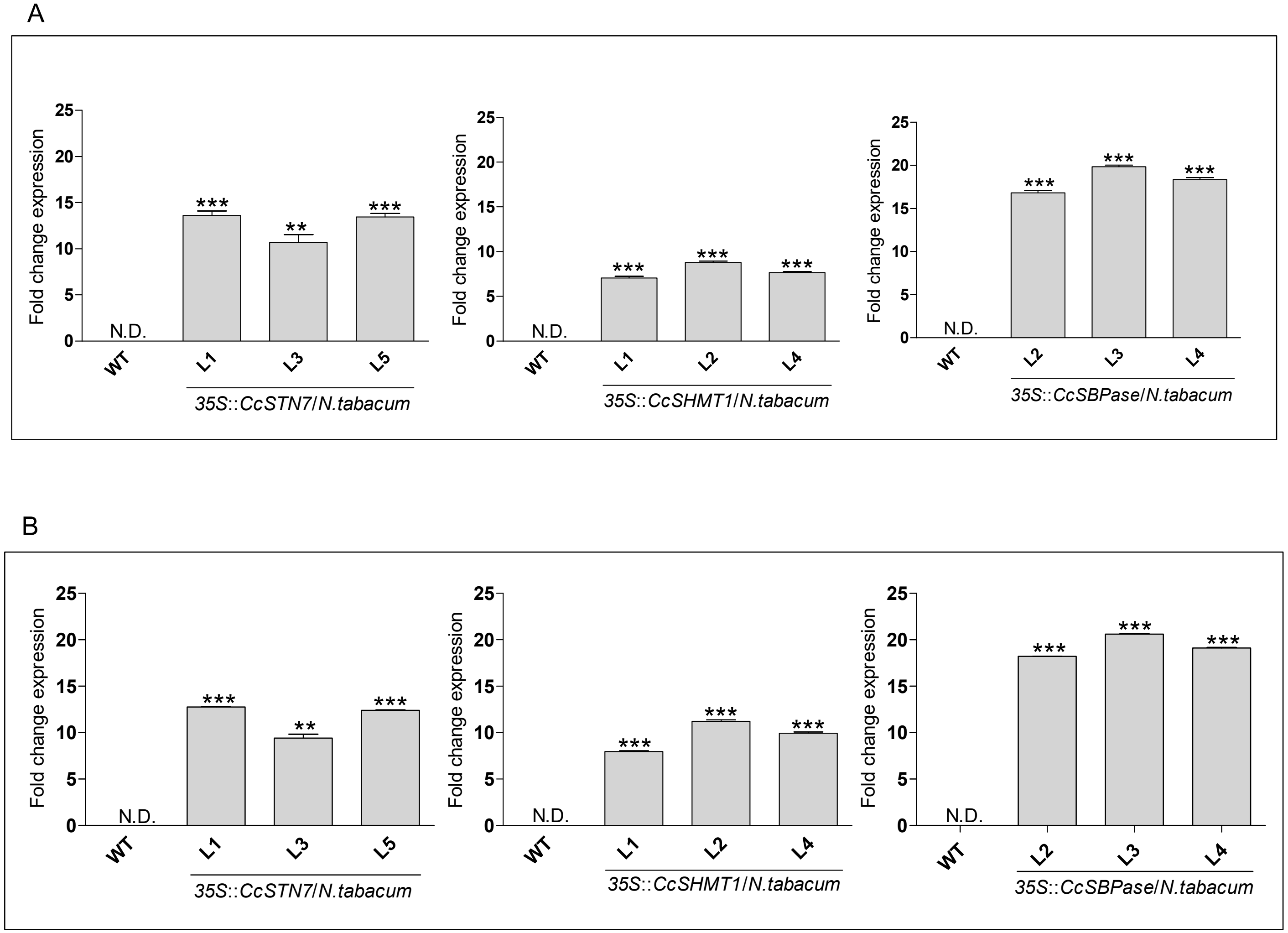

Figure S1. The RT-qPCR results of T2 *N.tabacum* transgenic lines. (**A**) The fold change data normalized against *HIST2H3A*, and (**B**) normalized against *HSC70-1*. Note: WT-Wild Type; N.D.- Not detected; Lines with asterisks (*) indicate a significant difference at P<0.05. A paired student t-test was used to compare the means of each transgenic line to WT.

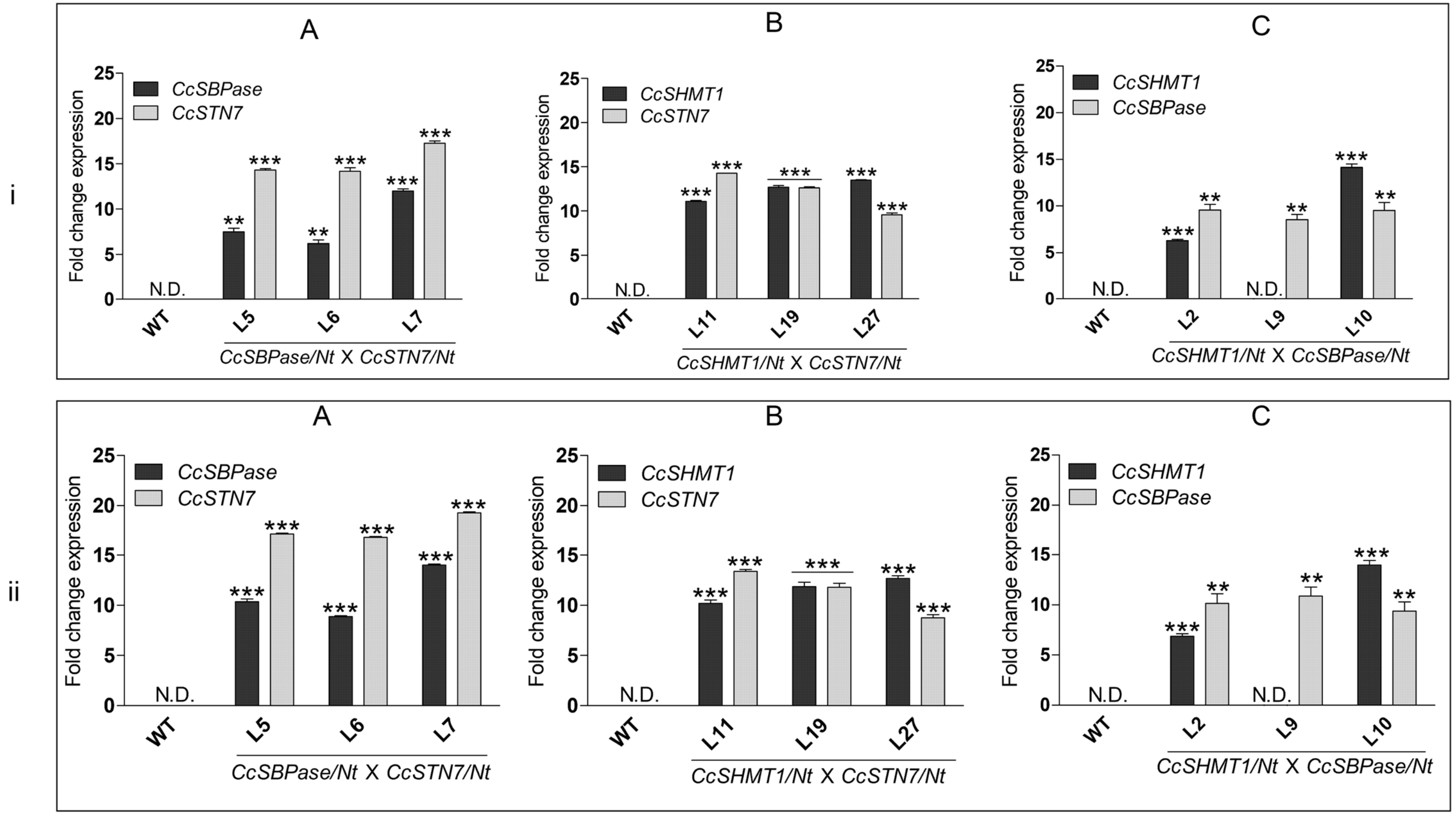

Figure S2. A RT-qPCR representation of F2 generation. (i) The fold change data normalized against the *HIST2H3A* reference gene, and (ii) normalised against *HSC70-1* for each crossing event. Note: WT-Wild Type; N.D.- Not detected; Lines with asterisks (*) indicate a significant difference at P<0.05. A paired student t-test was used to compare the means of each transgenic line to WT.

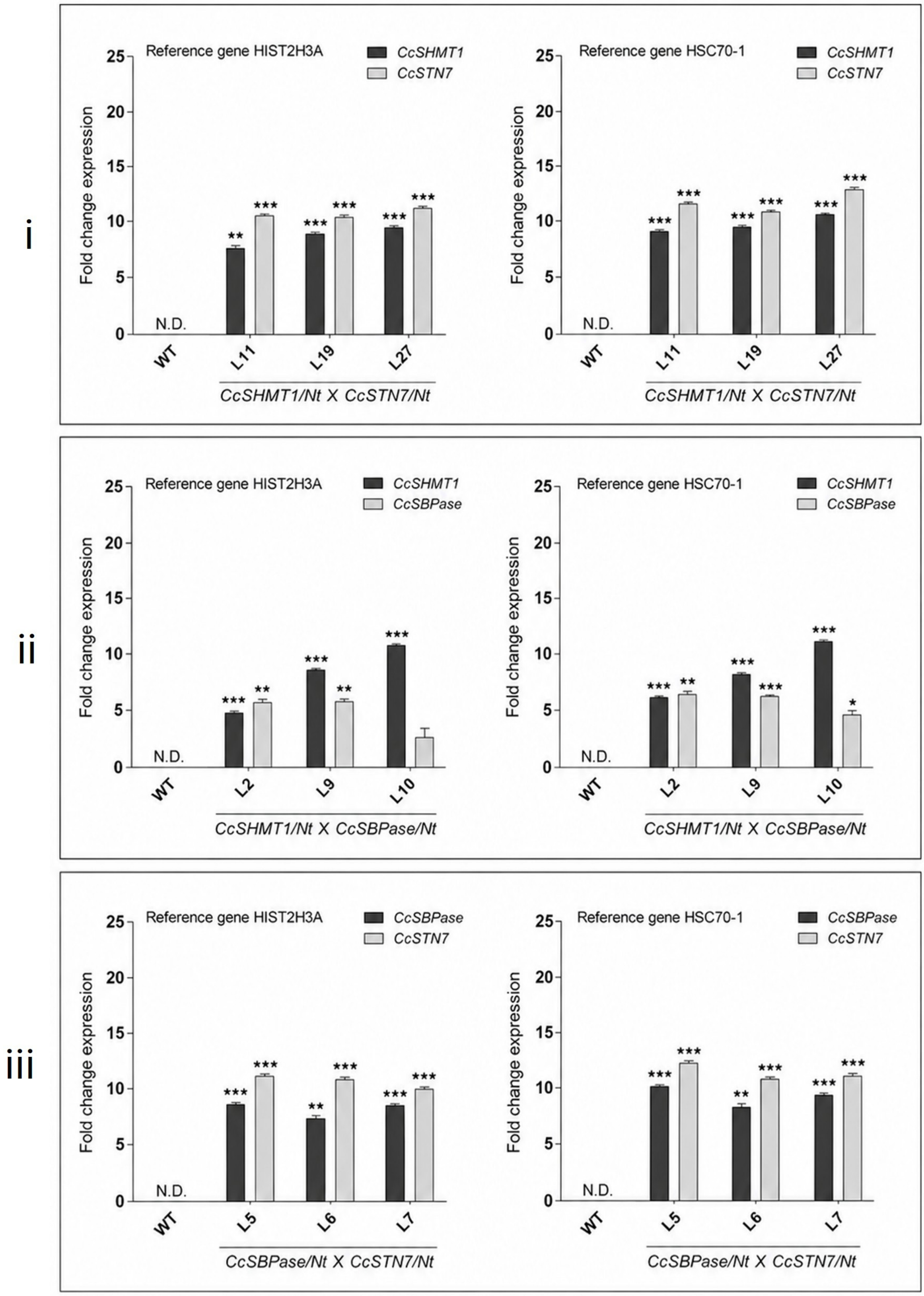

Figure S3. A RT-qPCR representation of the *N.tabacum* F1 generation resulting from a cross between (**i**) *35S*::*CcSHMT1*/*N.tabacum* L4(♀) X T2 *35S*::*CcSTN7*/*N. tabacum* L3(♂); (**ii**) *35S*::*CcSHMT1*/*N.tabacum* L2(♀) X L6 of F1 CcSBPase X CcSTN7 (♂) ; and (**iii**) *35S*::*CcSBPase*/*N.tabacum* Line 2(♀) X *35S*:: *CcSTN7*/*N.tabacum* Line 3(♂). The data is log2 fold change expression normalized against three reference genes. Note: WT-Wild Type; N.D.- Not detected; Lines with asterisks (*) indicate a significant difference at P<0.05. A paired student t-test was used to compare the means of each transgenic line to WT.
